# MAPK15 protects from oxidative stress‐dependent cellular senescence by inducing the mitophagic process

**DOI:** 10.1111/acel.13620

**Published:** 2022-06-01

**Authors:** Lorenzo Franci, Alessandro Tubita, Franca Maria Bertolino, Alessandro Palma, Giuseppe Cannino, Carmine Settembre, Andrea Rasola, Elisabetta Rovida, Mario Chiariello

**Affiliations:** ^1^ Istituto di Fisiologia Clinica (IFC) Consiglio Nazionale delle Ricerche (CNR) Siena Italy; ^2^ Core Research Laboratory (CRL) Istituto per lo Studio la Prevenzione e la Rete Oncologica (ISPRO) Siena Italy; ^3^ 9313 Department of Medical Biotechnologies University of Siena Siena Italy; ^4^ Department of Experimental and Clinical Biomedical Sciences University of Firenze Firenze Italy; ^5^ Department of Onco‐hematology, Gene and Cell Therapy Bambino Gesù Children’s Hospital–IRCCS Rome Italy; ^6^ Department of Biomedical Sciences University of Padova Padova Italy; ^7^ Telethon Institute of Genetics and Medicine (TIGEM) Pozzuoli Italy; ^8^ Department of Clinical Medicine and Surgery University of Napoli Federico II Napoli Italy

**Keywords:** autophagy, cellular senescence, MAP kinases, mitophagy, Oxidative DNA damage, signal transduction

## Abstract

Mitochondria are the major source of reactive oxygen species (ROS), whose aberrant production by dysfunctional mitochondria leads to oxidative stress, thus contributing to aging as well as neurodegenerative disorders and cancer. Cells efficiently eliminate damaged mitochondria through a selective type of autophagy, named mitophagy. Here, we demonstrate the involvement of the atypical MAP kinase family member MAPK15 in cellular senescence, by preserving mitochondrial quality, thanks to its ability to control mitophagy and, therefore, prevent oxidative stress. We indeed demonstrate that reduced MAPK15 expression strongly decreases mitochondrial respiration and ATP production, while increasing mitochondrial ROS levels. We show that MAPK15 controls the mitophagic process by stimulating ULK1‐dependent PRKN Ser^108^ phosphorylation and inducing the recruitment of damaged mitochondria to autophagosomal and lysosomal compartments, thus leading to a reduction of their mass, but also by participating in the reorganization of the mitochondrial network that usually anticipates their disposal. Consequently, MAPK15‐dependent mitophagy protects cells from accumulating nuclear DNA damage due to mitochondrial ROS and, consequently, from senescence deriving from this chronic DNA insult. Indeed, we ultimately demonstrate that MAPK15 protects primary human airway epithelial cells from senescence, establishing a new specific role for MAPK15 in controlling mitochondrial fitness by efficient disposal of old and damaged organelles and suggesting this kinase as a new potential therapeutic target in diverse age‐associated human diseases.

## Graphical abstract

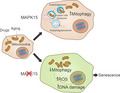

**Abbreviations**
ATP: adenosine tri‐phosphate
BAF: A1bafilomycin A1
DDR: DNA damage response
ECAR: Extracellular acidification rate
ETC: electron transport chain
FCCP: carbonyl cyanide 4‐(trifluoromethoxy) phenylhydrazone
GFP: green fluorescent protein
KD: kinase‐dead
LIR: LC3‐interacting region
MAP1LC3B/LC3B: microtubule‐associated protein 1 light chain 3
MAPK15: Mitogen‐activated protein kinase 15
mt‐DNA: mitochondrial DNA
MT‐ND: mitochondrially encoded NADH dehydrogenase
OCR: Oxygen consumption rate
OMM: outer mitochondrial membrane
OXPHOS: oxidative phosphorylation
PD: Parkinson's disease
ROS: Reactive oxygen species
SA‐β‐Gal: senescence‐associated β‐galactosidase
TOMM20: translocase of outer mitochondrial membrane 20
WT: wild‐type

## 1 INTRODUCTION

Besides representing the main source of adenosine tri‐phosphate (ATP) synthesis, mitochondria are also crucial in many other cellular processes such as apoptosis, necrosis, autophagy, stress regulation, intermediary metabolism, Ca^2+^ storage and innate immunity. Consequently, their dysfunctions are associated with many human diseases (Suomalainen & Battersby, 2018). Importantly, although the electron transport chain (ETC) is very efficient during oxidative phosphorylation (OXPHOS), approximately 1%–3% of mitochondrial oxygen consumed during this process is incompletely reduced, with ‘leaky’ electrons quickly interacting with molecular oxygen to form superoxide anions, the predominant reactive oxygen species (ROS) in mitochondria (Chance et al., 1979). Therefore, while small amounts of mitochondrial ROS (mt‐ROS) are a normal byproduct of OXPHOS, impairment or reduced efficiency in mitochondrial respiration induce an increased level of mt‐ROS, which, when not properly eliminated, may become highly toxic for the integrity of the cell (Kirkinezos & Moraes, 2001).

In physiological conditions, mitochondria are constantly kept under strict quality control. A marked mitochondrial dysfunction can activate organelle turnover by stimulating its degradation through a selective type of autophagy, a process named mitophagy, demonstrated to occur *in vivo* in many tissues both as a housekeeping, basal mechanism, and upon multiple stressful stimuli, that is, starvation, high fat diet, ischemia and hypoxia (Zachari & Ktistakis, 2020). Currently, the best studied mitophagy pathway is regulated by the Parkinson's disease (PD) proteins PTEN Induced Kinase 1 (PINK1) and Parkin (PRKN; PARK2). Importantly, while PINK1 phosphorylates PRKN on Ser^65^ when the latter reaches the mitochondria (Zachari & Ktistakis, 2020), an earlier ULK1‐dependent phosphorylation on PRKN Ser^108^ has been recently described to control mitophagy (Hung et al., 2021). An established consequence of altered mitophagy, and of the resulting increase of mt‐ROS, is cellular senescence (García‐Prat et al., 2016), a complex stress response by which proliferative cells permanently lose the ability to divide, exerting beneficial suppressive effects on the development of cancer and other proliferative diseases (Di Micco et al., 2021). However, depending on the context or specific pathogenetic stimuli, an excessive senescence can have also detrimental effects and cause or contribute to different aging‐associated phenotypes and diseases (Di Micco et al., 2021).

A role for the mitogen‐activated protein kinase 15 (MAPK15; ERK8; ERK7) protein, an atypical member of the MAP kinase family, has been recently shown in different mechanisms used by cells to manage stressful stimuli. Starvation, ionizing radiations or chemical insult due to cigarette smoke are all examples of stimuli requiring MAPK15 for mounting a defensive cellular response (Lau & Xu, 2018; Zhang et al., 2021). Particularly, starvation induces a complex series of events aimed at preserving cell survival under conditions of limited availability of nutrients, with MAPK15 participating in many starvation‐dependent cellular events among which Unc‐51 Like Autophagy Activating Kinase 1 (ULK1)‐dependent induction of autophagy (Colecchia et al., 2012, 2018). DNA damage also starts a complex cellular program directed to repair DNA lesions, and MAPK15 has been involved in cell mechanisms aimed at coping with different mutagenic sources (Klevernic et al., 2009; Li et al., 2018; Rossi et al., 2016), cooperating with proteins deeply involved in DNA repair or maintenance (Cerone et al., 2011; Groehler & Lannigan, 2010). Very recently, work on chronic obstructive pulmonary disease, a pathology already correlated to mitochondrial dysfunction and mitophagy‐dependent necroptosis, has suggested a role for MAPK15 in these events (Zhang et al., 2021). Importantly, we have already suggested a role for MAPK15 in intracellular pathways potentially able to control mitochondria physiology (Rossi et al., 2011). As mitochondria are the major intracellular source of ROS under normal physiological conditions (Kausar et al., 2018), we have investigated a potential role for MAPK15 in controlling oxidative stress‐dependent cellular senescence, by its ability to regulate the mitophagic process. Here, we demonstrate that mammal MAPK15 is a key modulator of mitochondrial homeostasis, capable of protecting primary as well as immortalized human cells from cellular senescence.

## 2 RESULTS

### 2.1 Mitochondrial respiration is altered in HeLa cells upon MAPK15 downregulation

**FIGURE 1:**
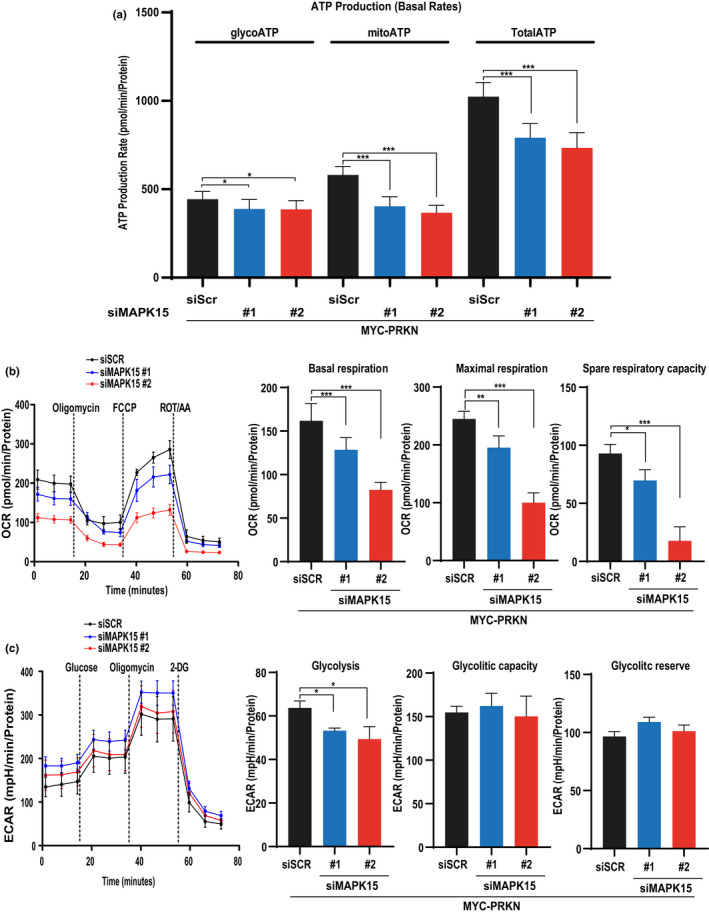
MAPK15 regulates ATP production rates. In all experiments, HeLa cells were transfected with scrambled siRNA or two different siRNA against MAPK15 (#1 and #2). After 24 h, they were also transfected with MYC‐PRKN and after additional 48 h, we proceeded to analysis. (a) Analysis of ATP production. Comparison of mitochondrial ATP (mitoATP) production rate and glycolytic ATP (glycoATP) production rate at basal level. (b) Analysis of oxygen consumption rate (OCR) was performed upon subsequent additions of oligomycin, FCCP and the respiratory complex I and III inhibitors rotenone and antimycin A, as indicated. (c) Analysis of the extracellular acidification rate (ECAR) with glycolysis stress test. Subsequent additions of glucose, the ATP synthase inhibitor oligomycin, and the hexokinase inhibitor 2‐deoxy‐glucose (2‐DG) were carried out as indicated. One experiment, representative of 3 independent experiments, is shown

Deregulation of homeostatic processes supervising mitochondria integrity and functional efficiency often manifests as alterations in the ability to produce ATP through OXPHOS. Therefore, we decided to evaluate the impact of MAPK15 deregulation on mitochondrial respiratory function, which can be readily examined using the Seahorse metabolic analyser. Importantly, we decided to use the HeLa cells to perform following experiments, as they do not express the PRKN gene (Strappazzon et al., 2015), which is usually necessary for an efficient mitophagic process (Zachari & Ktistakis, 2020), allowing us to rescue its expression by transfecting it, when necessary. HeLa cells expressing reduced levels of the MAPK15 gene by transient transfection of two specific and unrelated siRNAs (Figure S1) (Colecchia et al., 2015; Rossi et al., 2016) showed a severe decrease of total basal (i.e., in the absence of mitochondrial inhibitors) ATP production, that was mainly due to a reduced ability to generate ATP by OXPHOS (mitoATP) rather than to a decreased glycolytic production of ATP (glycoATP) (Figure 1a). Accordingly, evaluation of Oxygen Consumption Rate (OCR), an established measure of mitochondrial function, showed that basal respiration, an index of energetic demand of the cell under basal conditions, was strongly reduced in cells interfered for MAPK15 expression (Figure 1b). Interestingly, maximal respiration (i.e., the maximum rate of respiration that the cell can achieve) as well as spare respiratory capacity (i.e., an indicator of the capability of the cell to respond to energetic demand) were similarly affected upon MAPK15 knockdown (Figure 1b). Conversely, Extracellular acidification rates (ECAR), showed a small reduction of glycolysis following MAPK15 interference and no significant differences in glycolytic capacity (i.e., the maximum level of glycolysis that the cell can achieve) and glycolytic reserve (i.e., an indicator of the capability of the cell to respond to energetic demand) indicators (Figure 1c). Importantly, effects of MAPK15 deregulation on ATP production was not due to impairment of glucose uptake, as the amount of 2–2‐(*N*‐(7‐Nitrobenz‐2‐oxa‐1,3‐diazol‐4‐yl)Amino)‐2‐Deoxyglucose (2‐NBDG) was even increased in HeLa cells knocked down for MAPK15 expression (Figure S2). Overall, our results suggest that MAPK15 affects cellular ATP production primarily by acting on processes controlling homeostasis of the mitochondrial compartment.

### 2.2 MAPK15 counteracts the formation of mitochondrial ROS

**FIGURE 2:**
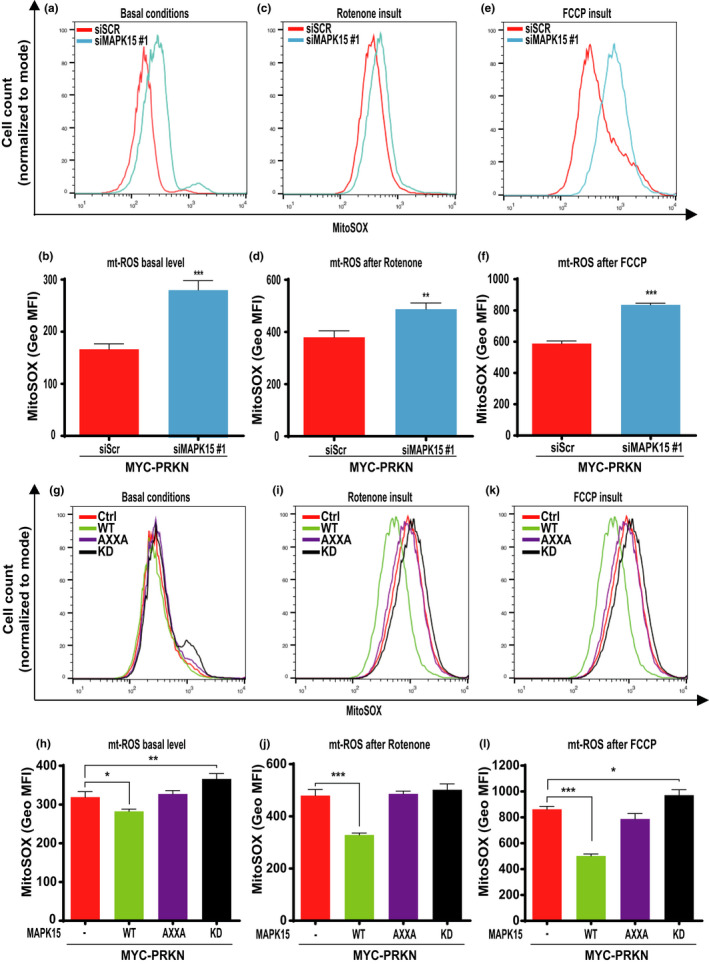
MAPK15 controls production of mt‐ROS. (a, c, e) Representative FACS histograms or (b, d, f) geometric mean fluorescent intensity (GeoMFI) of MitoSOX fluorescence, from HeLa cells transfected with scrambled siRNA or MAPK15 siRNA (#1) and, after 24 h, also transfected with MYC‐PRKN. After additional 48 h, samples underwent FACS analysis for MitoSOX red (5µM) fluorescence. Mitochondrial ROS were evaluated in basal conditions (a, b) and after Rotenone (4 h, 5 µM) (C, D), or FCCP (4 h, 30 µM) (e, f) insults. (g, i, k) Representative FACS histograms or corresponding (h, j, l) geometric mean fluorescent intensity (GeoMFI) Bars of MitoSOX fluorescence from HeLa cells transiently overexpressing MYC‐PRKN and empty vector (Ctrl) or MAPK15_WT or its mutant (AXXA, KD). Twenty‐four hours after transfection, samples underwent FACS analysis for MitoSOX red (5 µM) fluorescence. Mitochondrial ROS were evaluated in basal conditions (g, h) and after Rotenone (4 h, 5 µM) (I, J), or FCCP (4 h, 30 µM) (K, L) insults. Bars represents the standard deviation (SD) of 3 independent experiments (*n* = 3)

Reduced mitochondrial ATP production upon MAPK15 interference suggested us an impairment of mitochondrial respiration, which often determines increased levels of mt‐ROS (Kirkinezos & Moraes, 2001). Therefore, we investigated the impact of MAPK15 deregulation on mt‐ROS production. To this aim, we used the fluorogenic dye MitoSOX Red coupled to flow cytometry analysis. Specifically, we measured mt‐ROS both in unstimulated conditions and upon two different stimuli known to increase mt‐ROS by acting on mitochondrial targets, that is, rotenone, an inhibitor of mitochondrial complex I (Li et al., 2003) and the carbonyl cyanide 4‐(trifluoromethoxy) phenylhydrazone (FCCP), a protonophore uncoupler (Maro & Bornens, 1982). MAPK15 downregulation by siRNAs, in PRKN‐expressing HeLa cells, determined increased levels of mt‐ROS as compared to control cells, both in unstimulated conditions (Figure 2a,b) and upon induction of mitochondrial stress by rotenone (Figure 2c,d) or FCCP (Figure 2e,f), suggesting that MAPK15 functions are important to counteract superoxide production from mitochondria and contribute to their homeostasis. To reinforce these results, we next examined the effects of MAPK15 overexpression on mt‐ROS production, in PRKN‐expressing unstimulated or rotenone/FCCP‐treated HeLa cells. Overexpression of wild‐type (WT) MAPK15 determined a reduction of mt‐ROS in all conditions tested, compared to control (Figure 2g,h; Figure I‐L; Figure 2m,n), suggesting an increased capacity of these cells to counteract mt‐ROS production. Our laboratory has previously described a new LC3‐Interacting Region (LIR) motif in MAPK15 and engineered a mutated version of the kinase in this domain, MAPK15_AXXA, which is specifically deficient in its autophagic function (Colecchia et al., 2012). We, therefore, used this mutant to investigate whether the effects of MAPK15 on mitochondrial respiratory function and on mt‐ROS production might be ascribed to its ability to affect autophagy, possibly regulating the mitophagic process. Indeed, differently from what observed for MAPK15_WT, overexpression of the MAPK15_AXXA and MAPK15_KD mutants were not able to reduce mt‐ROS levels in unstimulated (Figure 2g,h) and rotenone‐ (Figure 2i‐l) or FCCP‐treated cells (Figure 2m,n). Overall, these data suggest that the autophagic function of this kinase is able to limit mt‐ROS superoxide production from mitochondria in basal conditions, for example, by controlling normal turnover of old organelles, but also to eliminate acutely damaged mitochondria, for example, upon treatment with agents such as rotenone and FCCP.

### 2.3 MAPK15 tightly controls the mitophagic process by inducing ULK1‐dependent PRKN phosphorylation

**FIGURE 3:**
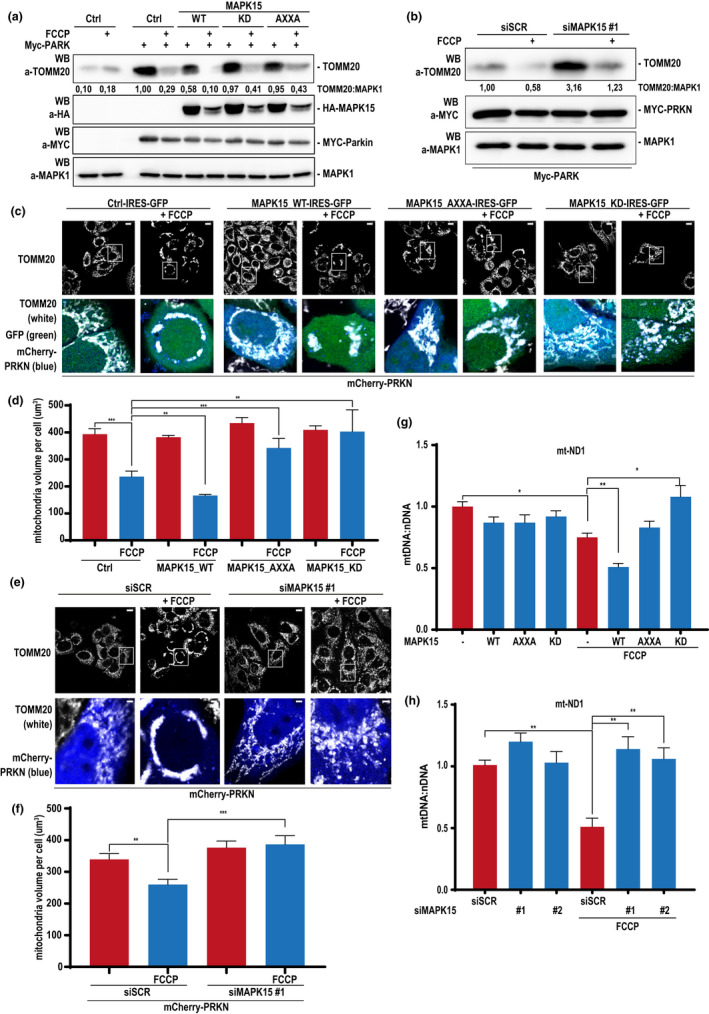
MAPK15 regulates mitochondrial volume and dynamics upon mitophagic stimuli. (a) HeLa cells were transfected with MYC‐PRKN and the empty vector (Ctrl) or MAPK15_WT or MAPK15_AXXA or MAPK15_KD. After 24 h, cells were treated with 30 µM FCCP or vehicle (8 h). Lysates were subjected to SDS‐PAGE followed by WB and analysed for indicated proteins. First two lanes do not express MYC‐PRKN. (b) HeLa cells were transfected with scrambled siRNA or MAPK15 siRNA (#1) and after 24 h, were transfected with MYC‐PRKN. After additional 48 h, samples were treated with 30 µM FCCP or vehicle (8 h). Lysates were then subjected to SDS‐PAGE followed by WB and analysed for indicated proteins. One experiment, representative of 3 independent experiments is shown. Densitometric analysis of bands is indicated. (c) HeLa cells were transfected with mCherry‐PRKN and Ctrl‐IRES‐GFP or MAPK15_WT‐IRES‐GFP or MAPK15_AXXA‐IRES‐GFP or MAPK15_KD‐IRES‐GFP. After 24 h, cells were incubated with 30 µM FCCP or vehicle (4 h). Cells were next fixed and subjected to immunofluorescence analysis. Scale bars correspond to 10 μm. (d) Mitochondria mean volume per cell, expressed in µm^3^. (e) HeLa cells were transfected with scrambled siRNA or MAPK15 siRNA (#1) and, after 24 h, further transfected with plasmid encoding for mCherry‐PRKN. After additional 48 h, cells were incubated 30 µM FCCP or vehicle (4 h). Cells were next fixed and subjected to immunofluorescence analysis. Scale bars correspond to 10 μm. (f) Mitochondria mean volume per cell, expressed in µm^3^. Bars represents the SD of 3 independent experiments (*n* = 3). (g) HeLa cells transiently overexpressing MYC‐PRKN and empty vector (Ctrl) or MAPK15_WT or MAPK15_AXXA or MAPK15_KD. Twenty‐four hours after transfection, samples were treated with 30 µM FCCP or vehicle (8 h). DNAs (10 ng) were subjected to qRT‐PCR for mitochondrially encoded NADH dehydrogenase 1 (MT‐ND1). The amount of PKM (pyruvate kinase M1/2), a nuclear‐encoded gene, was used for normalization purposes. (H) HeLa cells were transfected with scrambled siRNA or two MAPK15 siRNA (#1 or #2) and after 24 h, transfected with MYC‐PRKN. After additional 48 h, samples were treated with 30 µM FCCP or vehicle (8 h). DNAs (10 ng) were subjected a qRT‐PCR for MT‐ND1. The amount of PKM was used for normalization purposes. Bars represents average ratio ± SD between mitochondrial DNA and nuclear DNA (mt‐DNA:nDNA) of 3 independent experiments (*n* = 3)

Currently, there is no clear consensus within the scientific community with regard to a single optimal method for monitoring mitophagy (Klionsky et al., 2021). For this reason, several independent approaches are necessary to support the occurrence of this process (Klionsky et al., 2021). Among them, a key starting point for monitoring mitophagy is the analysis of the mitochondrial mass, to establish the efficiency of the process in eliminating dysfunctional organelles, aged or damaged by toxic substances (Klionsky et al., 2021). We, therefore, decided to evaluate, by western blot analysis, the levels of different mitochondrial proteins, whose changes are directly related to variations of mitochondrial mass, which can be consistently reduced by uncoupling agents such as FCCP (Gao et al., 2020). Indeed, TOMM20 (translocase of outer mitochondrial membrane 20) amounts were readily reduced upon FCCP treatment of PRKN‐expressing HeLa cells, while its levels increased further upon the same stimulus in cells that were not rescued for PRKN expression (Figure 3a, compare first and second lane to third and fourth, respectively). This result confirmed that, in our system, FCCP induced PRKN‐dependent mitophagic reduction of TOMM20 amounts, which therefore represented a good surrogate for mitochondrial mass. In this system, we transfected MAPK15_WT and its mutated counterparts, MAPK15_KD and MAPK15_AXXA, and demonstrated that the WT protein increased the efficiency in the elimination of mitochondria damaged by FCCP, as demonstrated by a reduction of TOMM20 protein levels, while both mutants failed to do so (Figure 3a). Correspondingly, downregulation of endogenous MAPK15 determined an increase of TOMM20 protein levels in PRKN‐expressing cells, in both FCCP‐stimulated and ‐unstimulated conditions (Figure 3b), demonstrating an accumulation of damaged mitochondria due to the lack of MAPK15. During mitophagy mediated by PRKN, this protein ubiquitinates a wide range of OMM components, including VDAC1, MFN1/2 and TOMM20, inducing their degradation by the proteasome (Klionsky et al., 2021). Estimating the amount of mitochondrial mass by using a single protein such as TOMM20 may, therefore, be deceiving (Geisler et al., 2010). Consequently, we decided to monitor four additional mitochondrial proteins with the same methodological approach, upon FCCP treatment, that is, ATP5A, UQCRC2, SDHB and NDUFB8, part of respiratory chain complexes V, III, II and I, respectively (Monzio Compagnoni et al., 2018). Indeed, upon reduction of endogenous MAPK15 in PRKN‐expressing HeLa cells, we observed increased levels of each of these proteins in basal conditions and upon FCCP stimulation (Figure S3), demonstrating accumulation of damaged mitochondria depending on MAPK15 downregulation and confirming TOMM20 as a good reporter for mitochondrial mass.

Confocal fluorescence microscopy is another powerful approach that can be used to score the amount of specific mitochondrial markers, to estimate the mass of these organelles (Klionsky et al., 2021). In addition, this approach also allows to get morphological information, which are becoming increasingly important to understand the regulation of homeostatic processes controlling the functions of mitochondria. Based on our data supporting the use of TOMM20 as a reliable endogenous surrogate for estimating mitochondrial mass, we visualized mitochondria by anti‐TOMM20 antibodies, in PRKN‐expressing HeLa cells transfected with bicistronic plasmids expressing green fluorescent protein (GFP) together with wild‐type (WT) and mutated forms (KD and AXXA) of MAPK15 (Colecchia et al., 2015). Specifically, in this experiment, we measured the mean volume of mitochondria per cell and showed that MAPK15_WT increased the efficacy of mitophagy after FCCP insult, as demonstrated by a reduction of mitochondrial volume compared to unstimulated cells (Figure 3c,d). Conversely, upon FCCP insult, both MAPK15_KD and MAPK15_AXXA determined an increase of mitochondrial volume (Figure 3c,d), demonstrating reduced capability of autophagy‐deficient MAPK15 mutants to support removal of damaged mitochondria. Interestingly, several authors have reported the observation of PRKN‐dependent mitochondrial clustering (sometimes called ‘mito‐aggresomes’), often in the perinuclear area, as a consequence of their loss of membrane potential (ΔΨ*_m_*), upon treatment with uncoupler drugs, such reorganization of the network usually anticipating their lysosomal degradation (Strappazzon et al., 2015; Vives‐Bauza et al., 2010). Indeed, we also noticed very pronounced perinuclear clusters of mitochondria, in PRKN‐expressing cells, upon prolonged (>2 h) exposure to FCCP, which were strongly prevented by co‐expression of both MAPK15_AXXA and MAPK15_KD mutants (Figure 3c), suggesting a specific role for this MAP kinase in the dynamics of the mitochondrial network involved in mitophagy (Vives‐Bauza et al., 2010). Ultimately, we decided to prove, also in these settings, that endogenous MAPK15 was able to control autophagic disposal of damaged mitochondria. We, therefore, downregulated MAPK15 by a specific siRNA in PRKN‐expressing HeLa cells and stimulated them with FCCP, to damage the mitochondrial compartment. As shown in Figure 3e,f, downregulation of MAPK15 expression prevented the ability of FCCP‐treated cells to eliminate mitochondria, which, on the contrary, was very efficient in cells expressing the endogenous MAP kinase. Strikingly, while mitochondria, upon FCCP treatment, changed their appearance from being primarily tubular and organized in an interconnected network throughout the cell body to clusters mostly in the perinuclear area (Vives‐Bauza et al., 2010), the same treatment barely affected the morphology and localization of these organelles in cells knockdown for endogenous MAPK15 expression (Figure 3e).

Quantitative PCR (qPCR) of specific mitochondrial genes may give a reliable estimation of mitochondrial DNA (mt‐DNA) copy number per cell and can be a useful alternative method to estimate mitochondrial mass (Klionsky et al., 2021). We, therefore, overexpressed MAPK15 and its mutated counterparts (KD and AXXA) in PRKN‐expressing HeLa cells, stimulated with FCCP, and next quantified mt‐DNA copy number by performing qPCR on two mitochondrial genes, MT‐ND1 (mitochondrially encoded NADH dehydrogenase1) and MT‐ND2 (Klionsky et al., 2021). In these conditions, FCCP reduced the quantity of these two genes and MAPK15_WT cooperated with this stimulus to further decrease their amounts (Figure 3g and Figure S4a). Conversely, upon FCCP treatment, MAPK15_AXXA could not affect mt‐DNA amount while MAPK15_KD even increased it (Figure 3g and Figure S4a), confirming a role for MAPK15 and its pro‐autophagic function in controlling mitophagy, upon mitochondrial damage. Ultimately, to confirm these results in a MAPK15‐endogenous setting, we also downregulated its levels by two specific and unrelated siRNA and demonstrated that reduced amounts of this MAP kinase strongly interfered with FCCP on the amounts of mt‐DNA (for both MT‐ND1 and MT‐ND2 genes) (Figure 3h and Figure S4b) and, consequently, with the efficacy of the mitophagic process induced by the protonophore uncoupler in these cells.

**FIGURE 4:**
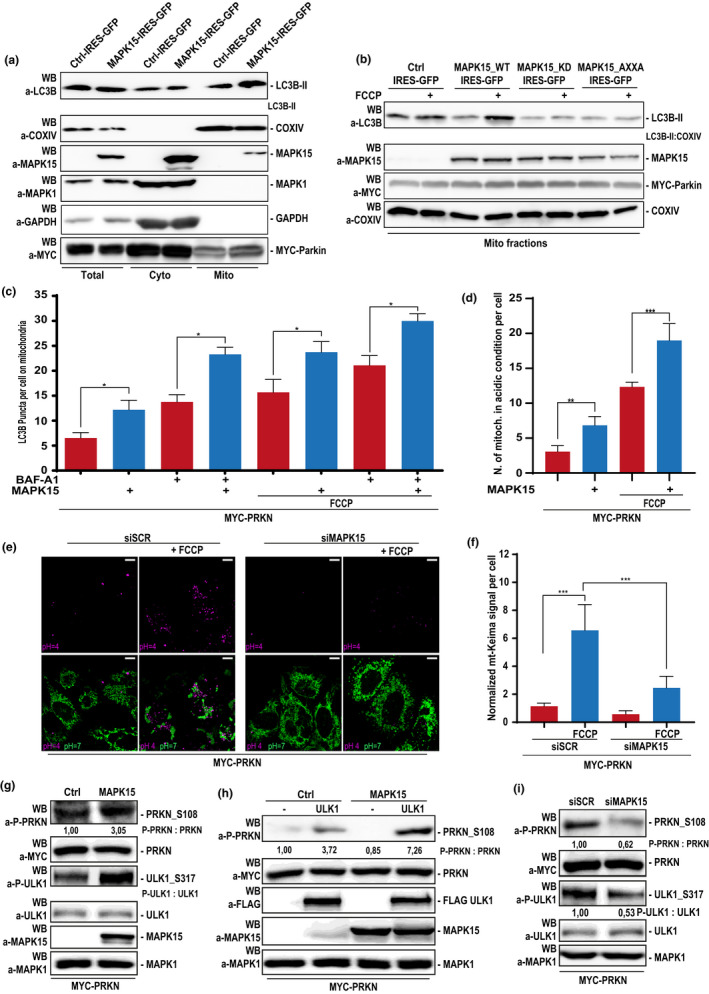
MAPK15 regulates mitophagy by controlling the extent of PRKN activating phosphorylation. (a) HeLa cells stably expressing the empty vector (Ctrl) or MAPK15_WT were transfected with MYC‐PRKN and, after 24 h, were subjected to fractioning obtaining mitochondrial enrichment. Lysates were analysed for indicated proteins. Total, total lysate; Cyto, cytoplasmic fraction; Mito, mitochondrial enrichment. One experiment, representative of 3 independent experiments is shown (*n* = 3). Densitometric analysis of bands is shown. (b) HeLa cells stably expressing the empty vector (Ctrl), MAPK15_WT, MAPK15_AXXA or MAPK15_KD were transfected with MYC‐PRKN and, after 24 h, were treated with 30 µM FCCP (1 h), where indicated, and then subjected to fractioning obtaining mitochondrial enrichment. Lysates were subjected to SDS‐PAGE followed by WB for analysis for indicated proteins. (C) HeLa cells stably expressing pDsRed2‐Mito were transfected with MYC‐PRKN and empty vector (Ctrl) or HA‐MAPK15 WT. After 24 h, cells were treated 1 h with vehicle or 30 µM FCCP or with 100 nM BAF‐A1 or 30 µM FCCP plus 100 nM BAF‐A1. Cells were next fixed and subjected to immunofluorescence analysis. LC3B dots per cell on mitochondria were plotted as result of five representative fields. Representative images are given in Figure S5. (d) Same as in (c), but cells were stained for endogenous LAMP1. Number of mitochondria in acidic condition were plotted, as result of five representative fields. Representative images are given in Figure S6. Bars represents the SD of 3 independent experiments (*n* = 3). (e) HeLa cells were transfected with scrambled siRNA or MAPK15 siRNA. After 24 h, they were next transfected with MYC‐PRKN and mt‐Keima. After additional 48 h, mitophagy was analysed by confocal microscopy. Mitochondria in neutral condition (pH = 7) are shown in green, while mitochondria in acidic condition (pH = 4) are shown in magenta. Scale bars correspond to 10 μm. (f) Intensitometric analysis of normalized Magenta/Green mt‐Keima signal per cell ± SD of seven different fields from the experiment in (e). (g) HeLa cells were transfected with MYC‐PRKN and the empty vector (Ctrl) or MAPK15_WT and, after 24 h were collected and lysates were subjected to SDS‐PAGE followed by WB. One experiment, representative of 3 independent experiments, is shown. Densitometric analysis of bands is shown. (h) HeLa cells were transfected with MYC‐PRKN, the empty vector (Ctrl) or MAPK15_WT and, where indicated, with FLAG‐ULK1. Lysates were subjected to SDS‐PAGE followed by WB and analysed for indicated proteins. One experiment, representative of 3 independent experiments is shown. Densitometric analysis of bands is shown. (i) HeLa cells were transfected with scrambled siRNA or siRNA against MAPK15 (#1) and after 24 h, were transfected with MYC‐PRKN. After additional 48 h, lysates were subjected to SDS‐PAGE followed by WB. One experiment, representative of 3 independent experiments is shown. Densitometric analysis of bands is shown

Induction of mitophagy can also be monitored by studying recognition of mitochondria by autophagosomes, detecting the enrichment of the LC3B protein in the mitochondrial fraction by immunoblot analysis (Klionsky et al., 2021). To this aim, we used PRKN‐expressing HeLa cells stably overexpressing MAPK15. In line with the hypothesis of a role for MAPK15 in the mitophagic process, we observed an increased amount of LC3B‐II in the mitochondrial fraction, (Figure 4a), demonstrating increased recruitment of autophagosomes to mitochondria, a key event in the initiation of mitochondrial disposal through mitophagy (Strappazzon et al., 2015). Interestingly, MAPK15 was also present in the mitochondrial fraction (Figure 4a), a localization possibly mediated by protein‐protein interactions, since specific mitochondrial targeting sequences are absent in this MAP kinase (e.g., http://busca.biocomp.unibo.it/deepmito/). To establish a functional role for MAPK15 in the mitophagic process by this approach, we also took advantage of HeLa cells stably overexpressing mutated forms of MAPK15, including above‐mentioned mutants lacking kinase (MAPK15_KD) or autophagic (MAPK15_AXXA) activities. Interestingly, mitochondrial fractions showed increased LC3B‐II amount in cells expressing the wild‐type protein while displayed a reduction of its levels in those expressing kinase‐ and autophagy‐deficient mutants, both in basal conditions and upon stimulation with the mitophagy inducer FCCP (Figure 4b). These data establish a clear dependency of the initial phases of the mitophagic process on the correct functioning of the MAPK15 protein.

Another key step for clear demonstration of the occurrence of the mitophagic process is the evidence of increased levels of autophagosomes containing or interacting with mitochondria. Therefore, we studied, by confocal microscopy, the effect of MAPK15 overexpression on mitochondria colocalization with puncta marked by LC3B (MAP1LC3B, microtubule‐associated protein 1 light chain 3), in unstimulated conditions and after a mitophagic stimulus, that is, FCCP. Importantly, this approach was coupled to bafilomycin A1 (BAF‐A1) treatment, to perform flux analysis (Klionsky et al., 2021). MAPK15 overexpression in PRKN‐expressing HeLa cells significantly augmented the number of LC3B puncta colocalizing with mitochondria, both in basal conditions (full medium) and upon 1‐hour FCCP stimulation, and this effect further increased upon BAF‐A1 treatment, indicating a positive mitophagic flux (Figure 4c and Figure S5). To complete the imaging analysis of the mitophagic flux, we next evaluated the fusion process of mitophagosomes with hydrolase‐containing lysosomes, which represents the last step in the degradation process along the autophagic route (Klionsky et al., 2021). Again, we chose to study the effect induced by MAPK15 overexpression on the fusion of mitochondria with LAMP1‐marked lysosomes. Specifically, we counted the number of mitochondria colocalized with and engulfed in lysosomes in PRKN‐expressing HeLa cells, and demonstrated that MAPK15 increased the localization of mitochondrial structures inside the lysosomal vesicles both in basal conditions and after the FCCP mitophagic stimulus (Figure 4d and Figure S6). Ultimately, to support our previous results, we next directly measured mitophagy by a pH‐sensitive, mitochondrial matrix‐targeted fluorescent Keima reporter protein (mt‐Keima) (Sun et al., 2015). Supporting our previous results, downregulation of MAPK15 in PRKN‐expressing HeLa cells prevented the ability of FCCP‐treated cells to stimulate the mitophagic process, as demonstrated by a reduced localization of mt‐Keima‐targeted mitochondria to the autophagolysosome compartment (Figure 4e,f). Overall, all our different approaches clearly demonstrate that MAPK15 is necessary for efficient disposal of damaged mitochondria in mammalian cells.

The AMPK‐ULK1 signaling axis plays a key role in mitophagy (Iorio et al., 2021). Based on our previous demonstration of the ability of MAPK15 to control ULK1 activity (Colecchia et al., 2018) and on the recent observation that PRKN is a direct substrate for ULK1 itself (Hung et al., 2021), we next decided to define a possible mechanism by which MAPK15 may control PRKN activation, by inducing ULK1‐dependent PRKN Ser^108^ phosphorylation. Indeed, MAPK15 strongly induced phosphorylation of the ULK1 phosphorylation site on PRKN (Figure 4g) and potentiated PRKN Ser^108^ phosphorylation induced by ULK1 (Figure 4h) while, in turn, depletion of the endogenous MAP kinase readily reduced phosphorylation of the ULK1 phospho‐site on PRKN (Figure 4i), overall demonstrating the ability of MAPK15 to control PRKN activating phosphorylation.

### 2.4 MAPK15 prevents cellular senescence by protecting genomic integrity from mt‐ROS produced by damaged mitochondria

**FIGURE 5:**
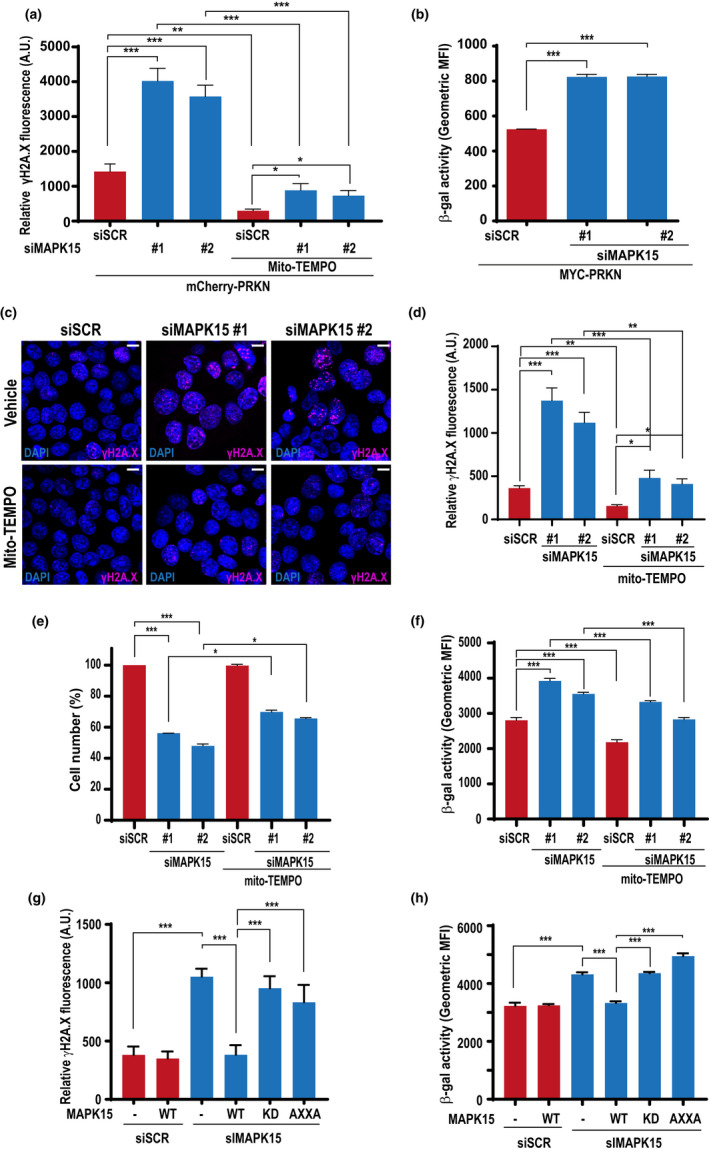
MAPK15 prevents DNA damage induced by mt‐ROS and controls cellular senescence in HeLa and SH‐SY5Y cells. (a) HeLa cells were transfected with scrambled siRNA or two different MAPK15 siRNA (#1 or #2). After 24 h, they were transfected with mCherry‐PRKN. After additional 24 h, cells were treated with 100 µM mito‐TEMPO or vehicle (24 h). Cells were next fixed and subjected to immunofluorescence analysis. Scale bars correspond to 7,5 μm. Intensitometric analysis of nuclear γH2A.X fluorescence of five representative microscopy fields. (b) HeLa cells were transfected with scrambled siRNA or two different MAPK15 siRNA (#1 or #2) and after 24 h, they were transfected also with MYC‐PRKN. After additional 48 h, cells were fixed and incubated with a probe staining senescent cells (β‐galactosidase activity). Then, they were analysed by FACS. Bars represents the SD of 3 independent experiments (*n* = 3). (c) SH‐SY5Y cells were transfected with scrambled siRNA or two different MAPK15‐specific siRNA (#1 or #2). After 48 h, cells were treated with 100 µM mito‐TEMPO or vehicle (24 h). Cells were next fixed and subjected to immunofluorescence analysis. Scale bars correspond to 10 μm. (d) Intensitometric analysis of nuclear γH2A.X fluorescence from five representative microscopy fields from experiment in (c). (e) SH‐SY5Y cells were transfected with scrambled siRNA or two different MAPK15 siRNA (#1 or #2) and after 48 h, they were treated with 100 µM mito‐TEMPO or vehicle (24 h). After 72 h of transfection, cells were harvested and cell number was evaluated with Z2 Coulter Counter (Beckman Coulter). (f) SH‐SY5Y cells were transfected with scrambled siRNA or two different MAPK15 siRNA (#1 or #2) and after 48 h, they were treated with 100 µM mito‐TEMPO or vehicle (24 h). Then, they were analysed by FACS. Bars represents the SD of 3 independent experiments (*n* = 3). (g) SH‐SY5Y cells were transfected with scrambled siRNA or siRNA specific for MAPK15 (#1 or #2), followed by rescue with ectopically expressed wild‐type (WT), autophagy‐deficient (AXXA) or kinase‐dead (KD) MAPK15. Control sample was transfected with the empty vector. Total nuclear γH2A.X signal was quantified in five representative fields using Volocity software and the graph represents intensitometric analysis of nuclear γH2A.X fluorescence. Bars represent SD of five representative microscopy fields. (h) Same as in (g), but cells were analysed by FACS for β‐Gal activity. Bars represents the SD of 3 independent experiments (*n* = 3)

Reduced efficacy in mitochondrial respiration increases generation of mt‐ROS which, in turn, damage several cellular components, including nuclear DNA, possibly contributing to senescence but also genomic instability and cancer (Di Micco et al., 2021). Based on the ability of MAPK15 to control mitophagy, we next asked whether ROS generated from mitochondria, as a consequence of decreased fitness, could be responsible for damaging nuclear DNA. To answer this question, we took advantage of a specific mitochondria‐targeted antioxidant, mito‐TEMPO (Porporato et al., 2014) to interfere with DNA damage induced by downregulation of MAPK15, scored as an increase in the levels of phosphorylated H2A histone family member X (γ‐H2A.X) foci. Indeed, mito‐TEMPO completely abolished DNA damage induced by MAPK15 knockdown, in PRKN‐expressing HeLa cells (Figure 5a and Figure S7), demonstrating that the increase in the production of mt‐ROS, due to impaired mitophagy, is the cause of this event so deleterious for the cell. Interestingly, increased levels of γ‐H2A.X foci and of ROS are frequently used as markers of cellular senescence (González‐Gualda et al., 2021). Therefore, to verify whether MAPK15 may affect the onset of this important stress response pathway, we next evaluated also the activity of the senescence‐associated β‐galactosidase (SA‐β‐Gal) (González‐Gualda et al., 2021). Indeed, in this model system, two specific and non‐correlated siRNAs for MAPK15 strongly increased SA‐β‐Gal activity (Figure 5b), ultimately supporting a role for MAPK15 in this process and in preserving proliferative potential of mammalian cells by maintaining the correct and efficient disposal of old and damaged mitochondria.

Next, we decided to confirm the suggested role of MAPK15 in protecting cells from senescence in a different model system expressing endogenous levels of PRKN, namely SH‐SY5Y cells (Van Humbeeck et al., 2011). Indeed, also in these cells, downregulation of MAPK15 (Figure S8) increased the amount of γ‐H2A.X foci and this effect was readily reversed by treating the cells with mito‐TEMPO (Figure 5c,d). MAPK15 RNA interference also strongly reduced SH‐SY5Y proliferative potential (Figure 5e) and increased SA‐β‐Gal activity (Figure 5f), both phenotypes being readily reversed by treating the cells with mito‐TEMPO, overall demonstrating a causative role in cellular senescence for mt‐ROS, accumulating as a consequence of impaired mitophagy caused by dysregulated MAPK15. Notably, a siRNA‐resistant MAPK15_WT cDNA, ectopically expressed in MAPK15‐silenced cells, was able to completely rescue siRNA‐induced DNA damage (Figure 5g) and SA‐β‐Gal activity (Figure 5h) while both siRNA‐resistant corresponding mutants (MAPK15_AXXA and MAPK15_KD) failed to do so, further supporting the specificity of our knockdown approach. As an additional control to confirm the senescent phenotype in response to MAPK15 downregulation, we ultimately demonstrated a strong increase, in SH‐SY5Y cells, in the expression of the p21 protein (Figure S9a) and of different cytokines associated to senescent‐associated secretory phenotype (SASP) (Jochems et al., 2021) (Figure S9b), and in the appearance of telomere‐associated DNA damage foci (TAF) (Fumagalli et al., 2012; Hewitt et al., 2012) (Figure S9c), indicating that, in different model cell lines, MAPK15 is able to confer protection from oxidative stress‐dependent cellular senescence with a mechanism depending on its mitophagic function.

### 2.5 MAPK15 protects primary hAEC from undergoing cellular senescence

**FIGURE 6:**
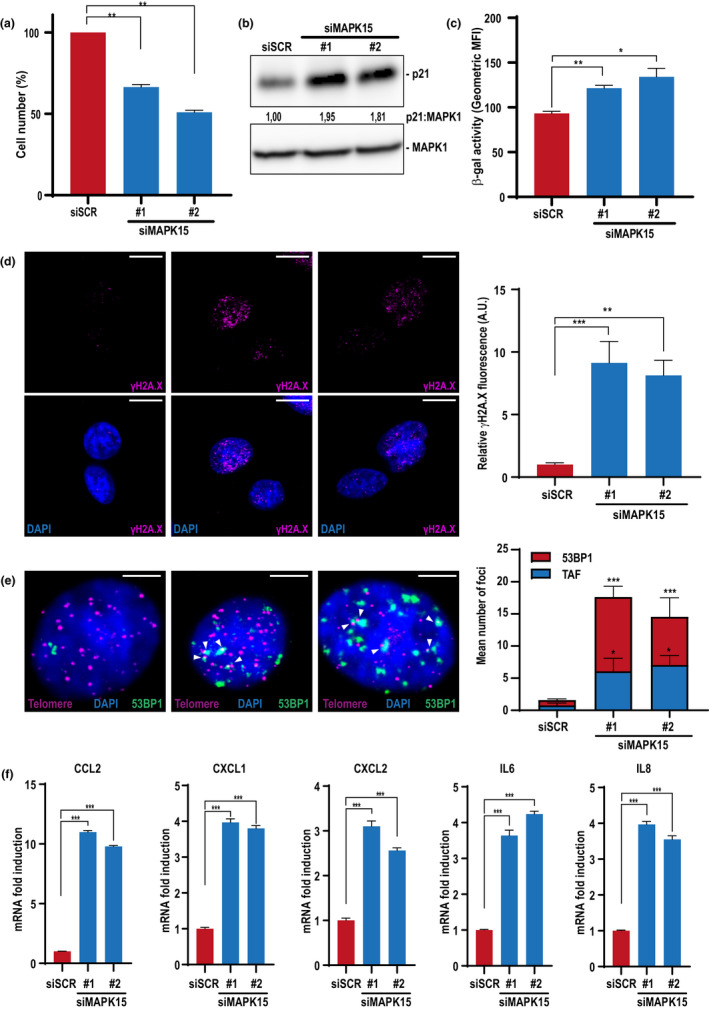
Reduced MAPK15 expression triggers cellular senescence in primary human Airway Epithelial Cells (hAEC). (a) hAEC cells were transfected with scrambled siRNA or two different MAPK15 siRNA (#1 or #2) and, after 72 h, they were harvested and cell number was evaluated with Z2 Coulter Counter (Beckman Coulter). (b) Same as in (a), but cells were lysed and subjected to WB analysis with indicated antibodies. (c) Same as in (a), but cells were fixed and incubated with a probe staining senescent cells (β‐galactosidase activity). Then, they were analyzed by FACS. (d) Same as in (a), but cells were fixed and subjected to immunofluorescence analysis with indicated antibody. Scale bars correspond to 10 μm. The accompanying graph shows intensitometric analysis of nuclear γH2A.X fluorescence from five representative microscopy fields. (e) Same as in (a), but cells were subjected to immuno‐FISH analysis. Representative images of DNA damage colocalizing with telomere (magenta, telomere; green, 53BP1; blue, nuclei) where white arrows indicate colocalization between telomeres and 53BP1. The accompanying graph shows the mean number of 53BP1 foci (red) and the colocalization between telomeres and 53BP1 (blue) per cell, >100 cells were analysed. Scale bars correspond to 5 μm. (f) Same as in (a), but cells were collected and subjected to qRT‐PCR to monitor mRNA expression of different cytokines associated to senescent‐associated secretory phenotype (SASP)

Ultimately, we decided to demonstrate the ability of MAPK15 to protect also primary cells from cellular senescence, using human Airway Epithelial Cells (hAEC), by testing several major markers of cellular senescence (González‐Gualda et al., 2021). Indeed, also in these non‐immortalized cells, downregulation of MAPK15 with two unrelated siRNA (Figure S10) reduced cell proliferation (Figure 6a), while increasing p21 protein levels (Figure 6b), SA‐β‐Gal activity (Figure 6c), the number of γ‐H2A.X foci (Figure 6d), the appearance of TAF (Figure 6e), and the expression of different cytokines associated to SASP (Figure 6f), ultimately demonstrating that a reduction of MAPK15 protein levels triggers cellular senescence in non‐immortalized human cells.

## 3 DISCUSSION

Our findings demonstrate that MAPK15 prevents the triggering of the cellular senescence phenotype by controlling the homeostasis of the mitochondrial compartment. Specifically, we show that this MAP kinase stringently regulates the mitophagic process from its very initial phases, by inducing ULK1‐dependent activating phosphorylation of PRKN. Still, our data also suggest that MAPK15 might control mitochondrial dynamics, possibly participating to mechanisms that segregates damaged organelles in the perinuclear area (Vives‐Bauza et al., 2010), therefore, not limiting its role only to speed up the mitophagic process but also possibly coordinating it to eventually spare intact mitochondria. Indeed, formation of these ‘mito‐aggresomes’ in the perinuclear area, a consequence of mitochondrial damage and a prerequisite for their disposal, is a microtubule‐dependent event (Okatsu et al., 2010) and the ability of FCCP of specifically inducing a reorganization of the cytoskeletal networks, particularly in HeLa cells, has been clearly established since long time (Maro & Bornens, 1982). Our observation that MAPK15 downregulation prevents formation of such structures upon FCCP treatment, suggests that this kinase may regulate the dynamics of the cytoskeleton to control mitophagy as well as other processes, a possibility that surely warrants further investigation.

Importantly, we show that MAPK15 may also be localized to mitochondria, possibly by interacting with proteins specifically recruited to these organelles (e.g., ULK1) upon mitophagic stimuli, suggesting that our observation of a reduction of MAPK15 protein levels upon FCCP treatment may be due to degradation, together with other mitochondrial resident proteins, during the mitophagic process. Ultimately, we show that the role of this kinase in mitophagy has also important biological implications, as a reduction of its expression induced an increase in the amount of mt‐ROS which caused extensive DNA damage and reduced cell proliferation of knocked down cells, all established markers of increased cellular senescence.

As generation of ROS is a byproduct of cell growth, cancer cells sustain a much higher level of ROS production compared to normal cells (Trachootham et al., 2006). Therefore, to avoid the damaging effects of oxidative stress, it is believed that cancer cells must actively upregulate multiple antioxidant systems. Among them, autophagy contributes to clear cells of all irreversibly oxidized biomolecules and damaged mitochondria, therefore, representing a fine mechanism to eliminate both the source and the consequences of oxidative stress, ultimately protecting cancer cells from oxidative damage. Therefore, it is believed that cancer cells strongly depend on these mechanisms for survival, and that their inhibition may represent a potential therapeutic approach to take advantage of specific tumor vulnerabilities (Luo et al., 2009). In this context, we have already shown both *in vitro* and *in vivo* that overexpression of MAPK15 gives a proliferative advantage to tumor cells (Colecchia et al., 2015; Rossi et al., 2016). It is tempting to speculate that this effect may be attributable, at least in part, to its ability to counteract endogenous oxidative stress and, possibly, oncogene‐induced senescence (Di Micco et al., 2021).

Our observation that, in different cell types, the mito‐TEMPO mitochondria‐targeted antioxidant strongly reduces nuclear damage and cellular senescence provoked by MAPK15 downregulation implies a strong contribution of this kinase to mechanisms able to determine the mutagenic load of the cells. Still, we must emphasize that MAPK15 has been already involved also in other mechanisms controlling DNA damage. Among them, it is interesting to notice that this MAP kinase sustains the activity of TERT/Telomerase (Cerone et al., 2011) an enzyme deeply involved in DNA‐damage response (DDR) and in opposing cellular senescence (Di Micco et al., 2021), again pointing to this MAP kinase as a key regulator of DNA damage, by controlling multiple proteins contributing to this process. Indeed, MAPK15 expression and activity are finely regulated by DNA damaging stimuli (Klevernic et al., 2009), therefore, suggesting the existence of a stress regulatory loop involving this kinase, possibly controlling specific aspects of the senescent phenotype, as already demonstrated also for MAPK14 (Passos et al., 2010). Importantly, even in the absence of exogenous chemical or physical insults such as FCCP, MAPK15 downregulation reduces basal mitophagy of ‘aged’ mitochondria and is sufficient to determine a strong increase in mt‐ROS levels and resulting nuclear DNA damage, supporting a key role for this kinase in the ‘housekeeping’ control of the elimination of the most dangerous source of mutagenic agents inside the cells, represented by old and damaged mitochondria, eventually leading to chronic DNA damage, a prominent cause of cellular senescence (Di Micco et al., 2021).

Eukaryotic cells are constantly subjected to changes of their micro‐environmental conditions. Based on this, they continuously need to adapt to such changes to optimize their survival strategies. A key role in the response to such potentially harmful situations is based on the concerted action of different cell response pathways, whose role is either to allow cells to adapt to novel external conditions or to commit them to suicidal mechanisms, when excessively damaged. Data from our, as well as from other laboratories, now indicate MAPK15 as an important regulator of different adaptive response pathways, often interconnected among each other's, suggesting a potential major role for MAPK15 in integrating responses to such stimuli, which often determine important human pathologies. Importantly, dysfunctions in the mitophagic process have been linked to many pathological conditions, often related to aging, with neurodegenerative diseases such as PD probably representing the best examples (Yoo & Jung, 2018). Intriguingly, *in vivo* depletion of senescent cells in a PD mouse model also determines a reduction in the development of corresponding neurodegeneration (Chinta et al., 2018), suggesting that strategies aimed at reducing an already established senescence phenotype may represent a successful approach to cure PD affected individuals. Overall, our current demonstration of the role of MAPK15 in supporting mitochondrial fitness suggests that approaches targeting this kinase may allow the modulation of cell senescence in humans, to possibly increase its beneficial effects in aggressive neoplastic diseases or reduce its detrimental effects in PD and other age‐related disorders.

## 4 EXPERIMENTAL PROCEDURES

### 4.1 Expression vectors

pCEFL‐HA‐MAPK15 and all its mutants were already described (Colecchia et al., 2012; Iavarone et al., 2006). pCEFL‐MAPK15_WT IRES‐GFP and all its mutants (AXXA; KD) were previously described (Colecchia et al., 2015). MAPK15_WT, MAPK15_AXXA and MAPK15_KD mutants resistant to siRNA for MAPK15 were previously described (Rossi et al., 2016). pRK5‐Myc‐Parkin (MYC‐PRKN) (Addgene #17612), FLAG‐ULK1 (Addgene #24301) and mCherry‐Parkin (mCherry‐PRKN) (Addgene #23956), pHAGE‐mt‐mKeima (mt‐Keima) (Addgene #131626) were provided by Addgene as the kind gifts from the producing laboratories. The pDsRed2‐Mito plasmid was purchased from Clontech (Cat. #63242).

### 4.2 Statistical analysis

LC3B‐dots count, mitochondria in acidic conditions, mitochondrial volume, fluorescence intensity and telomere‐associated DDR foci (TAF) were analysed using the Quantitation Module of Volocity software (PerkinElmer Life Science, I40250). Significance (p value) was assessed by either pairwise Student's *t*‐test or multiple comparisons were analysed with one‐way ANOVA with post‐hoc Tukey's HSD (Honestly Significant Difference) multiple comparison, using GraphPad Prism8 software. Asterisks were attributed as follows: *p < 0.05, **p < 0.01, ***p < 0.001.

## CONFLICTS OF INTEREST

No potential conflicts of interest were disclosed.

## AUTHOR CONTRIBUTIONS

LF planned and run all the biochemical, molecular and cellular biology experiments and analysed the data; AT contributed to Seahorse experiments and helped in analysing the data; GC helped with Seahorse methodology; AR and ER supervised Seahorse experiments; AP and CS contributed to the mt‐Keima assay; MC conceived the study, analysed the experimental data and wrote the manuscript. All authors revised the manuscript.

## Supporting information

Supplementary Material

## Data Availability

Data sharing not applicable to this article as no datasets were generated or analysed during the current study.
