# Supplementary material for: MAPK15 protects from oxidative stress‐dependent cellular senescence by inducing the mitophagic process
Source: Aging Cell. 2022 Jun 1;21(7):e13620. doi: 10.1111/acel.13620 (PMC9282834; doi:10.1111/acel.13620)
Supplement: Supplementary file 1 — Supplementary Material [file ACEL-21-e13620-s001.docx]

**SUPPLEMENTARY MATERIAL**

**MAPK15 Protects from Oxidative Stress-Dependent Cellular Senescence by inducing the mitophagic process**

Lorenzo Franci^1,2,3^, Alessandro Tubita^4^, Franca Maria Bertolino^1,2^, Alessandro Palma^5^, Giuseppe Cannino^6^, Carmine Settembre^7,8^, Andrea Rasola^6^, Elisabetta Rovida^4^ and Mario Chiariello^1,2,^*

^1^Istituto di Fisiologia Clinica (IFC), Consiglio Nazionale delle Ricerche (CNR), Siena, Italy; ^2^Core Research Laboratory (CRL), Istituto per lo Studio, la Prevenzione e la Rete Oncologica (ISPRO), Siena, Italy; ^3^Department of Medical Biotechnologies, University of Siena, Siena, Italy; ^4^Department of Experimental and Clinical Biomedical Sciences, University of Firenze, Firenze, Italy; ^5^Department of Onco-hematology, Gene and Cell Therapy, Bambino Gesù Children’s Hospital–IRCCS, Rome, Italy; ^6^Department of Biomedical Sciences, University of Padova, Padova, Italy; ^7^Telethon Institute of Genetics and Medicine (TIGEM), Pozzuoli, Italy; ^8^Department of Clinical Medicine and Surgery, University of Napoli Federico II, Naples, Italy.

***Corresponding author:** Mario Chiariello, email: [mario.chiariello@cnr.it](mailto:mario.chiariello@cnr.it); Address: Istituto di Fisiologia Clinica (IFC), Consiglio Nazionale delle Ricerche CNR) and Core Research Laboratory (CRL), Istituto per lo Studio, la Prevenzione e la Rete Oncologica (ISPRO), Siena, Italy.

**SUPPLEMENTARY EXPERIMENTAL PROCEDURES**

**Reagents and antibodies**

Bafilomycin A1 (Alfa Aesar) was used at final concentration of 100 nM; Hoechst 33342 and 4',6–Diamidino-2-fenilindolo diidrocloruro (DAPI) (from VWR) were used at final concentration of 0.01 mg/mL and 3µM respectively; Carbonylcyanide-4-(trifluoromethoxy)-phenylhydrazone (FCCP) (Enzo Life Sciences) was used at final concentration of 30 µM. MitoSOX™ Red Mitochondrial Superoxide Indicator, for live-cell imaging (Thermo Fisher Scientific); 2-(N-(7-Nitrobenz-2-oxa-1,3-diazol-4-yl)Amino)-2-Deoxyglucose (2-NBDG) (Thermo Fisher Scientific); (2-(2,2,6,6-Tetramethylpiperidin-1-oxyl-4-ylamino)-2-oxoethyl)triphenylphosphonium chloride (Mito-TEMPO) (Sigma Aldrich); CellEvent Senescence Green Flow Cytometry Assay Kit (Thermo Fisher Scientific); DMEM, no glucose, no glutamine, no phenol red (Thermo Fisher Scientific). Seahorse XF Cell Mito Stress Test Kit, Glycolysis Stress Test Kit and Real-Time ATP Rate Assay Kit were purchased from Agilent Technologies. The following primary antibodies were used for western blots, confocal microscopy experiments: anti-MAPK15/ERK8 (custom preparation), anti-HA (Covance, MMS-101R) anti-LC3B (Nanotools, 0231-1000), anti-LC3B (MBL, M152-3), anti-MAPK1/ERK2 (Santa Cruz Biotechnology, sc-154), anti-LAMP1 (Santa Cruz Biotechnology, sc-20011), Total OXPHOS Rodent WB Antibody Cocktail (abcam ab110413), anti-Tomm20 (F-10) (Santa Cruz Biotechnology, sc-17764), anti-COX IV (4D11-B3-E8) (Cell Signaling, 11967), γH2A.X (Cell Signaling, 9718), Phospho-Parkin (Ser108) (Cell Signaling, 36728), Phospho-ULK1 (Ser317) (Cell Signaling, 12753), ULK1 (Cell Signaling, 8054), p21 (Epitomics, 3733-1), 53BP1 (Novus, NB100-304). The following secondary antibodies were used for western blot experiments: anti-mouse (Santa Cruz Biotechnology, sc-2004) and anti-rabbit (Santa Cruz Biotechnology, sc-2005) HRP-conjugated IgGs.

**Cell culture and transfections**

SH-SY5Y cells were maintained in Dulbecco’s modified Eagle medium (DMEM) F12 supplemented with 10% fetal bovine serum (FBS), 2 mM L-glutamine and 100 units/ml penicillin-streptomycin at 37°C in an atmosphere of 5% CO_2_/air. HeLa cells were maintained in Dulbecco’s modified Eagle medium (DMEM) supplemented with 10% fetal bovine serum (FBS), 2 mM L-glutamine and 100 units/ml penicillin-streptomycin at 37°C in an atmosphere of 5% CO_2_/air. Primary human airway epithelial cells (hAEC) were maintained in hAEC medium (Epithelix) at 37°C in an atmosphere of 5% CO_2_/air. HeLa Empty vector IRES-GFP, HA-MAPK15_WT IRES-GFP, HeLa HA-MAPK15_KD IRES-GFP, HA-MAPK15_AXXA IRES-GFP and HeLa pDsRed2-Mito were generated by transfecting HeLa cells with corresponding plasmids and then subjected to selection with 2 mg/ml G-418. Western blot for overexpression experiments, 3×10^5^ cells were seeded in 6-well cell culture plates and transfected with 1 μg of each expression vector using Lipofectamine LTX (Life Technologies, Monza, Italy), according to manufacturer’s instructions. For western blot with interference experiments, 1×10^5^ cells were seeded in 6-well cell culture plates, transfected with 100 nM (HeLa cells) or 200 nM (SH-SY5Y cells) of each siRNA with Hiperfect (Qiagen), according to the manufacturer’s instructions, for hAEC cells interference experiments RNAiMAX and 100 nM of each siRNA were used, according to the manufacturer’s instructions. Then cells were harvested after 72hrs. The co-overexpression with pRK5-Myc-PRKN or mCherry-PRKN were performed 24 hrs after siRNA transfection using Lipofectamine LTX (Life Technologies, Monza, Italy). For confocal microscopy experiments, 5×10^4^ cells were seeded on coverslips placed onto 12-well plates. Each sample was transfected with 200 ng of each plasmid using Lipofectamine LTX and then, 1×10^5^ cells were seeded in 6-well cell culture plates for confocal microscopy experiments and transfected with 100 nM (HeLa cells) or 200 nM (SH-SY5Y cells) of each siRNA with Hiperfect (Qiagen), and 100 nM of each siRNA with RNAiMAX for hAEC cells experiments, according to the manufacturer’s instructions. Twenty-four hours after transfection with siRNA, cells were transfected with mCherry-PRKN, where indicated, using Lipofectamine LTX (Life Technologies, Monza, Italy), according to manufacturer’s instructions. Forty-eight hours after transfection with siRNA, cells were trypsinized and seeded on coverslips placed onto 12-well cell culture plates at the concentration of 5×10^4^ cells/well. Fixation and staining with specific antibodies were performed 72 hrs after transfection.

**Western blots**

Total lysates were obtained by resuspending cellular pellets in RIPA buffer (50 mM TRIS-HCl pH 8.0, 150 mM NaCl, 0.5% sodium deoxycholate, 0.1% SDS, 1% NP-40) with the addition of protease inhibitors (cOmplete Protease Inhibitor cocktail, EDTA-free; Roche Diagnostics, Monza, Italy) and phosphatase inhibitors (2 mM NaF, 2 mM Na_3_VO_4_; Sigma Aldrich). Total proteins were quantified by Bradford assay and the same quantity of lysates was used for western blot analysis. Laemmli Loading Buffer 5X (250 mM Tris-HCl pH 6.8, 10% SDS, 50% glycerol, bromophenol blue) was added to protein samples, which were then heated for 5 min at 95°C or 50°C for when using Total OXPHOS Rodent WB Antibody Cocktail. Lysates were loaded on SDS-PAGE poly-acrylamide gel, transferred to Immobilon-P PVDF membrane (Merck Millipore), probed with appropriate antibodies, and revealed by enhanced chemiluminescence detection (ECL Plus; GE Healthcare, Milan, Italy). Densitometric analysis of western blots was performed with NIH Image J (National Institutes of Health).

**Immunofluorescence**

Cells were washed with PBS, fixed with ice-cold methanol for 10 min, and incubated with γH2A.X specific primary antibody for 1 hr. Alternatively, they were fixed with 4% paraformaldehyde in PBS for 20 min and permeabilized with 0.2% Triton X-100 solution for 5 min or 100 μg/ml digitonin solution (Invitrogen, BN2006) for 20 min for visualizing LC3B, then 20 min with 1% BSA in PBS. After blocking, cells were incubated with appropriate primary antibodies for 1 hr, washed three times with PBS, and then incubated with appropriate Alexa Fluor 488-conjugated (Invitrogen, A21202) or Alexa Fluor 647-conjugated (Invitrogen, A21245) secondary antibodies and then washed again three times in PBS. Nuclei were stained with 1.5 μM 4′,6-diamidino-2-phenylindole (DAPI) in PBS for 5 min. Coverslips were mounted in fluorescence mounting medium (Dako, S3023). Samples were visualized on a TSC SP5 confocal microscope (Leica, 5100000750) installed on an inverted LEICA DMI 6000CS (10741320) microscope using an oil immersion PlanApo 40× 1.25 NA objective or an oil immersion PlanApo 63× 1.4 NA objective. Images were acquired using the LAS AF acquisition software (Leica Microsystems).

**Mitochondrial DNA copy number quantitation and analysis of gene expression**

Total RNA was purified using QIAzol LysisReagent (Qiagen). Reverse transcription was performed with the QuantiTect Reverse Transcription Kit (Qiagen). Total DNA was purified using QIAamp DNA Mini Kit and loaded 10 ng of purified DNA on each RT-PCR reaction. RT-PCR was performed with Luna Universal qPCR Master Mix on a Rotor-Gene 6000 RT-PCR system (Corbett Life Science). The following primer pairs were used:

MT-ND1 FW 5’-GGCTATATACAACTACGCAAAGGC-3’;

MT-ND1 RV 5’-GGTAGATGTGGCGGGTTTTAGG-3’;

MT-ND2 FW 5’-CTTCTGAGTCCCAGAGGTTACC-3’;

MT-ND2 RV 5’-GAGAGTGAGGAGAAGGCTTACG-3’;

PKM FW 5’-ATGGCTGACACATTCCTGGAGC-3’;

PKM RV 5’-CCTTCAACGTCTCCACTGATCG-3’;

MAPK15 FW 5’-TGGCCAGCGTACAACAGGT-3’;

MAPK15 RV 5’-CAGTCCCGTAGGCTTGGGAGTA-3’;

MAPK1 FW 5’-GCCCATCTTTCCAGGGAAGCATTA-3’;

MAPK1 RV 5’-AGAGCTTTGGAGTCAGCATTTGGG-3’;

CCL2 FW 5’-GATCTCAGTGCAGAGGCTCG-3’;

CCL2 RV 5’-TGCTTGTCCAGGTGGTCCAT-3’;

CXCL1 FW 5’-GAAAGCTTGCCTCAATCCTG-3’;

CXCL1 RV 5’-CTTCCTCCTCCCTTCTGGTC-3’;

CXCL2 FW 5’-GGGCAGAAAGCTTGTCTCAA-3’;

CXCL2 RV 5’-GCTTCCTCCTTCCTTCTGGT-3’;

LIF FW 5’- CCCATCACCCCTGTCAACG-3’;

LIF RV 5’-GGGCCACATAGCTTGTCCA-3’;

IL6 FW 5’-GCAGAAAAGGCAAAGAATC-3’;

IL6 RV 5’-CTACATTTGCCGAAGAGC-3’;

IL8 FW 5’-GTTTTTGAAGAGGGCTGAG-3’;

IL-8 RV 5’-TTTGCTTGAAGTTTCACTGG-3’.

**Seahorse Analysis**

Cells were seeded in XFe96 cell culture plates with 2 × 10^4^ cells per well and after 24h subjected to the extracellular flux (XF). For the XF glycolysis stress test, XF assay medium was supplemented with 2 mM glutamine and incubated at 37 °C in a non-CO_2_ incubator for 1 hr before the analysis. This analysis is performed in real-time by measuring ECAR and OCR after Glucose (10mM) oligomycin (1.5 μM) and 2-DG (50 mM) administration. For the XF Mito Stress test and Real-Time ATP Rate XF base medium were supplemented with 10 mM glucose, 2 mM glutamine and 1 mM sodium pyruvate. Cells were incubated for 1h at 37 °C in a non-CO_2_ incubator before the analysis. This analysis is performed by real-time measurement of ECAR and OCR after a sequence of compounds, for the Mito Stress test oligomycin were used (1.5 μM), carbonyl cyanide-4 (trifluoromethoxy) phenylhydrazone (FCCP) (1 μM) and Rotenone/Antimycin A (0.5 μM). For the XF Real-Time ATP Rate were used oligomycin (1.5 μM) and Rotenone/Antimycin A (0.5 μM). Protein quantification was used to normalize the results. Results were analyzed using Wave 2.6 desktop software.

**2-NDBG Glucose Uptake**

Cells (2 x10^4^) were seeded in 96-well plate and, after 24 hrs, cells were washed with PBS, and incubated with glucose and FBS free medium containing 0.02 mg/mL of 2-NDBG (a fluorescent glucose analogue that can be accumulated inside the cells but cannot be processed) for 1 hr at 37°C and 5%CO_2_/air. After incubation, we washed cells with PBS and detected fluorescence with SpectroMax M2 microplate reader (Molecular Devices). Each well fluorescence was normalized by protein quantitation.

**Cells proliferation**

Cells (1×10^5^) were seeded in 6-well plates, then subjected to siRNA transfection. After 48 hrs, cells were treated with vehicle or mito-TEMPO (100 μM) for 24 hrs. After 72 hrs from transfection, cell number were evaluated using Z2 Coulter Counter (Beckman Coulter).

**Immuno-FISH**

Cells were grown on coverslips and immunofluorescence was performed as describe above with anti-53BP1. Cells were then fixed with methanol: acetic acid (3:1), dehydrated through an alcohol series, air-dried and incubated with PNA hybridization mix [70% deionized formamide (SIGMA), 250 uM MgCl2, 10 mM Tris pH 7.4, 0,5% blocking reagent (Roche) containing 400nM of Cy3-labelled telomere-specific (CCCTAA) peptide nucleic acid probe (Panagene)], for 10 min at 80°C and then for 1 hr at RT. Cells were washed twice with 2x SSC 0,1% TWEEN 20 for 10 min at 55°C. Cells were counterstained with 1.5 μM 4′,6-diamidino-2-phenylindole (DAPI) in 2x SSC for 10 min. Coverslips were mounted in fluorescence mounting medium (Dako, S3023).

**Knock-down of endogenous MAPK15**

MAPK15-specific siRNA #1 (MAPK15 #1; target sequence 5’-TTGCTTGGAGGCTACTCCCAA-3’) and control non-silencing siRNA (Scramble, target sequence 5’-AATTCTCCGAACGTGTCACGT-3’) were obtained from Qiagen. MAPK15-specific siRNA #2 (MAPK15 #2; target sequence 5’- GACAGAUGCCCAGAGAACATT -3’) was obtained from Eurofins Genomics. All siRNAs were transfected at a final concentration of 100 nM (HeLa, hAEC) or 200 nM (SH-SY5Y) using Hiperfect (Qiagen). Samples were collected 72 hrs after transfection.

**Mitochondrial ROS production**

The MitoSOX™ Red fluorescent probe (Molecular Probes, Inc., Eugene, OR, USA) is able to accumulate in mitochondria due to their positive charge, and its oxidation by superoxide produces red fluorescence that can be easily measured {Kauffman et al., 2016, #61360}. To quantify mitochondrial ROS production we incubated, 5 µM of MitoSOX with cells in full medium for 10 minutes, according to the manufacturer’s protocol. Samples were acquired on a FACSCanto II flow cytometer (BD Biosciences). Data were analyzed with FlowJo software. All analyses were performed in triplicate.

**Mitochondrial enrichment**

Cells were homogenized in RLM buffer (250 mM sucrose, 10 mM Tris–HCl [pH 7.5], 0,1 mM EGTA, 1 mM dithiothreitol, 2mM Na_3_VO_4_, 2 mM NaF) with the addition of protease inhibitors. Homogenates were centrifuged at 1000×g for 10 min at 4°C, the pellet were used as total fraction and the supernatant were collected and centrifuged at 6000×g for 10 min at 4°C. The supernatant was designated the cytosolic fraction, and the pellet was used as the mitochondrial enriched fraction. The pellets were resuspended in lysis buffer (20 mM HEPES, pH=7.5, 10 mM EGTA, 40 mM β-glycerophosphate, 1% NP-40, 2.5 mM MgCl_2_, 2 mM orthovanadate, 2 mM NaF, 1 mM DTT, Roche protease inhibitors cocktail). Then, samples were subjected to protein quantification and western blot analysis.

**β-galactosidase activity**

Senescence was monitored with fluorescence-based detection of β-galactosidase activity using the CellEvent Senescence Green Flow Cytometry Assay kit. HeLa cell line were subjected to siRNA transfection to knock down endogenous MAPK15 and after 24h, we overexpressed MYC-PRKN. Alternatively, we downregulated MAPK15 in SH-SY5Y, and 48 hrs after, cells were treated with mito-TEMPO (100 μM), where indicated, for 24 hrs. hAEC cells were subjected to siRNA knock down of endogenous MAPK15. After 72 hrs of siRNA transfection, we fixed cells in 4% paraformaldehyde in PBS for 20 min. Then samples, were incubated with CellEvent Senescence Green Probe at 37°C for 2 hours in the absence of CO_2_ following manufacturer’s instructions (Thermo Fisher Scientific). Samples were subjected to flow cytometer analysis, acquired with FACSCanto II cytometer (BD Biosciences).

**mt-Keima mitophagy assay**

HeLa cells (1x10^5^) were seeded on 35 mm coverglass #0 chamber dishes (MatTek), in DMEM containing 10% FBS supplemented with penicillin-streptomycin, L-glutamine, without phenol red. Then samples were subjected to transfection of scrambled and MAPK15 siRNA. After 24 hrs, we co-transfected MYC-PRKN and mt-Keima plasmids and, after additional 48 hrs, samples were incubated, where indicated, with 30 µM FCCP to induce mitophagy. Fluorescence of mt-Keima was imaged via two sequential excitations (458 nm, green; 594 nm, magenta) and emission was collected with a long pass filter with 605- to 695-nm emission range. Images were obtained taking z-stacks, and laser power was set at the lowest output that would allow clear visualization of the mt-Keima signal. Imaging settings were maintained with the same parameters for comparison between different experimental conditions. Calculation of mitophagy based on mt-Keima signal was performed employing the original images using Volocity software. Mitophagy was measured dividing the volume of magenta high intensity signal by the volume of green signal. The mitophagy was normalized by the number of cells and expressed as fold induction.

**SUPPLEMENTARY FIGURES**

**Figure S1.** Efficacy of endogenous MAPK15 knockdown by specific siRNA in HeLa cells.

**Figure S2.** Glucose uptake in MAPK15 knockdown cells.

**Figure S3.** MAPK15 regulates the abundance of different endogenous mitochondrial proteins.

**Figure S4.** MAPK15 controls mt-DNA elimination after mitophagic stimuli.

**Figure S5.** MAPK15 controls fusion of mitochondria to autophagosomes.

**Figure S6.** MAPK15 controls fusion of mitochondria to lysosomes.

**Figure S7.** MAPK15 prevents DNA damage induced by mt-ROS in HeLa cells.

**Figure S8.** Efficacy of endogenous MAPK15 knockdown by specific siRNA, in SH-SY5Y cells.

**Figure S9.** MAPK15 knockdown induces cellular senescence in SH-SY5Y cells.

**Figure S10.** Efficacy of endogenous MAPK15 knockdown by specific siRNA, in hAEC primary cells.

**Figure S1.** Efficacy of endogenous MAPK15 knockdown by specific siRNA in HeLa cells. HeLa cells were transfected with scrambled siRNA or two different MAPK15 siRNA (#1 or #2) and, after 24 hrs, they were transfected also with MYC-PRKN. Seventy-two hours after siRNA transfection, cells were collected and subjected to qRT-PCR to monitor mRNA expression (**a**) or to western blot analysis for to monitor endogenous MAPK15 protein levels (**b**).

**Figure S2.** Glucose uptake in MAPK15 knockdown cells. Hela cells were transfected with scrambled siRNA or two different MAPK15 siRNA (#1 or #2) and, after 24 hrs, they were transfected also with MYC-PRKN. Seventy-two hours after siRNA transfection, cells were treated with 2-NDBG (1 hr, 0.02 mg/mL), in FBS- and glucose- free medium, and detected fluorescence with a spectrophotometer. Bars represent the average ± SD Relative fluorescence Unit (RFU) of 2-NDBG fluorescence.

**Figure S3.** MAPK15 regulates the abundance of different endogenous mitochondrial proteins. HeLa cells were transfected with scrambled siRNA or MAPK15 siRNA (#1) and after 24h, were transfected with MYC-PRKN. After additional 48 hrs, samples were treated with 30 µM FCCP or vehicle (8 hrs). Lysates were then subjected to SDS-PAGE followed by WB and analyzed for indicated proteins. Densitometric analysis of bands is indicated.

**Figure S4.** MAPK15 controls mt-DNA elimination after mitophagic stimuli. (**a**) HeLa cells transiently overexpressing MYC-PRKN and empty vector (Ctrl) or MAPK15_WT or MAPK15_AXXA or MAPK15_KD. Twenty-four hours after transfection, samples were treated with 30 µM FCCP or vehicle (8 hrs). DNA was purified using QIAamp DNA Mini Kit and then 10 ng of each sample were subjected to qRT-PCR for mitochondrially encoded NADH dehydrogenase 2 (MT-ND2). The amount of PKM (pyruvate kinase M1/2), a nuclear-encoded gene, was also quantified by qPCR and used for normalization purposes. (**b**) HeLa cells were transfected with scrambled siRNA or two MAPK15 siRNA (#1 or #2) and after 24h, transfected with MYC-PRKN. After additional 48 hrs, samples were treated with 30 µM FCCP or vehicle (8 hrs). DNA was next purified using QIAamp DNA Mini Kit, and then 10 ng of each sample were subjected a qRT-PCR for MT-ND2. The amount of PKM was also quantified by qPCR and used for normalization purposes. Bars represents average ratio ± SD between mitochondrial DNA and nuclear DNA (mtDNA:nDNA). In each case, bars represent the SD of 3 independent experiments (n = 3).

**Figure S5.** MAPK15 controls fusion of mitochondria to autophagosomes. Analysed fields correspond to those showed in figure 3A. HeLa cells stably expressing pDsRed2-Mito were transfected with MYC-PRKN and empty vector (Ctrl) or HA-MAPK15 WT. After 24 hrs, cells were treated with vehicle or 30 µM FCCP or with 100 nM BAF A1 or 30 µM FCCP plus 100 nM BAF A1 together (1 hr). Cells were next fixed and subjected to immunofluorescence analysis. In these representative images, LC3B is visualized in green, HA-MAPK15 in blue and DsRed-Mito in red. Scale bars correspond to 7.5 μm.

**Figure S6.** MAPK15 controls fusion of mitochondria to lysosomes. Analysed fields correspond to those showed in figure 3B. HeLa cells stably expressing pDsRed2-Mito were transfected with MYC-PRKN and empty vector (Ctrl) or HA-MAPK15_WT. After 24 hrs, cells were treated with vehicle or 30 µM FCCP or with 100 nM BAF A1 or 30 µM FCCP plus 100 nM BAF A1 together (1 hr). Cells were next fixed and subjected to immunofluorescence analysis. In these representative images, LAMP1 is visualized in green, HA-MAPK15 in blue and DsRed-Mito in red. Scale bars correspond to 25 μm.

**Figure S7.** MAPK15 prevents DNA damage induced by mt-ROS in HeLa cells. HeLa cells were transfected with scrambled siRNA or two different MAPK15 siRNA (#1 or #2). After 24 hrs, they were transfected with mCherry-PRKN. After additional 24 hrs, cells were treated with 100 µM mito-TEMPO or vehicle (24 hrs). Cells were next fixed and subjected to immunofluorescence analysis. Scale bars correspond to 7,5 μm.

**Figure S8.** Efficacy of endogenous MAPK15 knockdown by specific siRNA, in SH-SY5Y cells. Cells were transfected with scrambled siRNA or two different MAPK15 siRNA (#1 or #2). Seventy-two hours after siRNA transfection, cells were collected and subjected to qRT-PCR to monitor mRNA expression (**a**) and western blot analysis to monitor MAPK15 protein expression (**b**).

**Figure S9**. MAPK15 knockdown induces cellular senescence in SH-SY5Y cells. (**a**) Cells were transfected with scrambled siRNA or two different MAPK15 siRNA (#1 or #2). Seventy-two hours after siRNA transfection, cells were lysed and subjected to WB analysis with indicated antibodies. (**b**) Same as in (**a**), but cells were collected and subjected to qRT-PCR to monitor mRNA expression of different cytokines associated to senescent-associated secretory phenotype (SASP). (**c**) SH-SY5Y after 72 hours of transfection with scrambled siRNA or two different MAPK15 siRNA (#1 or #2) were subjected to immuno-FISH. Representative images of DNA damage colocalizing with telomere (magenta, telomere; green, 53BP1; blue, nuclei) where white arrows indicate colocalization between telomeres and 53BP1. The accompanying graph shows the mean number of 53BP1 foci (red) and the colocalization between telomeres and 53BP1(blue) per cell, >100 cells were analysed. Scale bar represents 5µm.

**Figure S10.** Efficacy of endogenous MAPK15 knockdown by specific siRNA, in hAEC primary cells. Cells were transfected with scrambled siRNA or two different MAPK15 siRNA (#1 or #2). Seventy-two hours after siRNA transfection, cells were collected and subjected to qRT-PCR to monitor mRNA expression (**a**) and western blot analysis to monitor MAPK15 protein expression (**b**).
